# Diabetes patient management by pharmacists during Ramadan

**DOI:** 10.1186/1472-6963-14-117

**Published:** 2014-03-10

**Authors:** Kerry Wilbur, Kawthar Al Tawengi, Eman Remoden

**Affiliations:** 1 College of Pharmacy, Qatar University, PO Box 2713 Doha, Qatar

**Keywords:** Diabetes, Patient care, Fasting, Ramadan, Pharmacist

## Abstract

**Background:**

Many Muslim diabetes patients choose to participate in Ramadan despite medical advice to the contrary. This study aims to describe Qatar pharmacists’ practice, knowledge, and attitudes towards guiding diabetes medication management during Ramadan.

**Methods:**

A cross-sectional descriptive study was performed among a convenience sample of 580 Qatar pharmacists. A web-based questionnaire was systematically developed following comprehensive literature review and structured according to 4 main domains: subject demographics; diabetes patient care experiences; knowledge of appropriate patient care during Ramadan fasting; and attitudes towards potential pharmacist responsibilities in this regard.

**Results:**

In the 3 months prior to Ramadan (July 2012), 178 (31%) pharmacists responded to the survey. Ambulatory (103, 58%) and inpatient practices (72, 41%) were similarly represented. One-third of pharmacists reported at least weekly interaction with diabetes patients during Ramadan. The most popular resources for management advice were the internet (94, 53%) and practice guidelines (80, 45%); however only 20% were aware of and had read the American Diabetes Association Ramadan consensus document. Pharmacist knowledge scores of appropriate care was overall fair (99, 57%). Pharmacists identified several barriers to participating in diabetes management including workload and lack of private counseling areas, but expressed attitudes consistent with a desire to assume greater roles in advising fasting diabetes patients.

**Conclusion:**

Qatar pharmacists face several practical barriers to guiding diabetes patient self-management during Ramadan, but are motivated to assume a greater role in such care. Educational programs are necessary to improve pharmacist knowledge in the provision of accurate patient advice.

## Background

Ramadan is the holy month of fasting in which Muslims refrain from food, fluids, oral medications and smoking from sunrise until sunset. The Koran specifically exempts from fasting those for whom it may have harmful consequences, such as pregnant women, the elderly and those with medical conditions. Fasting is not recommended for type 1 diabetes patients, those who are non-adherent to therapy or who have poor glycemic control [1]. These individuals in particular are at increased risk of hypo- and hyperglycemic episodes as well as metabolic complications associated with dehydration [2]. However, many diabetes patients choose to participate despite the medical and religious advice excusing them from the Ramadan fast [3-5].

Fortunately, diabetes management strategies during Ramadan have been proposed by international medical and religious bodies [1,6-9]. General considerations include pre-Ramadan medical assessment and patient education regarding meal planning; physical activity; the need for increased frequency of blood glucose monitoring and the recognition and management of acute complications. Recommendations for diabetes medication dosing adjustments tailored for both type 1 and type 2 diabetes patients are also outlined. Despite availability of such resources, studies have identified gaps in prescribed diabetes patient care, notably inappropriate health professional discouragement towards fasting and lack of counseling regarding medications or instructions for breaking the fast [10-12].

Regions with the highest diabetes prevalence worldwide are currently found in Gulf Coast Countries (GCC) [13]. Qatar is an Arab emirate occupying a small peninsula in the Persian Gulf where estimates of diagnosed diabetes among its population of 1.9 million have ranged from 12% (all residents) to 17% (among Qatari only) [14,15]. Qatar boasts an emerging economy and is recently devoting significant resources to develop its health care infrastructure, with emphasis on primary health care, including enhancing roles of community pharmacy [16,17]. Provision of health and medication education associated with Ramadan is a vehicle for such expansion of pharmacist scope of practice and patient expectations thereof. Pharmacists, especially in ambulatory care practices, are easily accessible by patients and can assume an important role in ensuring safe fasting for diabetes patients; however, no studies on such pharmacist care have been reported. The objective of this study is to explore Qatar pharmacists’ current practice, knowledge and attitudes towards guiding diabetes patient management during Ramadan.

## Methods

A comprehensive English language literature review was conducted in pertinent electronic health databases (PubMed, EMBASE, IPA, CINAHL: 1990-January 2011) and Google and Google Scholar search engines using combination of predetermined key words, “*diabetes”;* “*Ramadan*”; “*fast*”; ““*pharmacist*”; “*attitudes*”; “*perceptions*”; “*experiences*”; “*knowledge*”; “*barriers”.* Online resources of multinational organizations, such as the World Health Organization and International Diabetes Federation were verified. Online resources were similarly searched using key words in Arabic. Hand-search of references of retrieved articles was also performed. Clinical practice guidelines, consensus statements, reviews or reports outlining diabetes patient management during Ramadan, and any research pertaining to health professional fasting diabetes patient care experience were evaluated and adapted for development of our questionnaire.

Survey questions were structured according to 4 main domains: subject demographics; diabetes patient care experiences; knowledge of appropriate patient care during Ramadan fasting; and attitudes towards potential pharmacist responsibilities in this regard. The section assessing respondent knowledge consisted of a series of questions related to recommendations for diabetes patient medical assessments and drug dosing derived from published clinical practice guidelines and evidence-based reviews [1,6-9]. Areas evaluated pertained to risks to diabetes patients during fasting (seven statements); patient risk stratification (twelve statements); recommendations for referral and evaluation (twenty nine statements); drug dosing adjustment (three statements); and safety parameters for prematurely breaking the fast (two statements). One mark was awarded for each appropriate answer, with a maximum obtainable correct score of 53. An arbitrary scoring system categorized overall level of knowledge assessed as poor (0–25), moderate (26–40) and good (41–53). A further twenty-seven statements addressing pharmacist attitudes towards perceived barriers (14) and opportunities (6), as well as motivations (7) for expanding roles in diabetes care management during Ramadan. A five-point Likert scale ranging from “Strongly Agree” to “Strongly Disagree” was used to assess the responses of the participants.

A draft survey was then reviewed for face and content validity by a small working group comprised of three senior pharmacist members of Qatar University (QU) College of Pharmacy (CPH) faculty and staff and two licensed pharmacists. Modifications were made based on feedback provided and consensus reached among all involved in revising the questionnaire.

The questionnaire was formatted as an electronic survey (SurveyMonkey^®^ program) and piloted by a small randomly selected group of pharmacists, after which no necessary design changes were made. Reliability testing was conducted for the attitude responses results showing internal consistency of the items tested with Cronbach’s α value of 0.75.

Qatar is an emerging economy but its infrastructure for health professionals lags behind that of developed countries. In the absence of a national pharmacy regulatory body, there is no complete database of pharmacists working in Qatar [18]. The Qatar University College of Pharmacy maintains an independent internal database of 580 pharmacist contacts which represents 75%-80% of all pharmacists practicing in the country. This convenience sample of pharmacists employed within documented public and private health care facilities were invited to participate in the survey.

Pharmacists were invited by email to participate in the anonymous web-based survey that remained open for response over a 3-month period prior to Ramadan in July 2012. Email reminders were sent at designated intervals at 2, 4, 6, and 8 and 10 weeks. The research proposal was approved by the QU Institutional Review Board (IRB).

Frequencies of correct answers to knowledge questions were assessed. To simplify analysis of the 5-point Likert scale employed for attitude questions, we classified those who answered “Strongly Agree” and “Agree” as having agreed and those who answered “Strongly Disagree” and “Disagree” as having disagreed. Categorical data is presented as percentages of frequency or occurrence and continuous data is presented as means with standard deviations. Responses were further stratified according to demographic parameters of practice setting, length of practice experience, frequency of diabetes patient interactions, and religious faith. Statistical comparisons of frequencies utilized χ^2^ -tests with alpha of less than or equal to 0.05 considered statistically significant. All data analyses was conducted using IBM SPSS^®^ for MAC release 20.0.

## Results

The survey remained open between April 8 and June 30, 2012. (3 months prior to Ramadan in July 2012). One hundred and seventy-eight pharmacists (31%) completed the questionnaire during this time. Seventeen different countries of origin were represented. Most had attained a Baccalaureate as the highest pharmacy degree (156, 87.6%) and over half of respondents had over a decade of experience (74, 54.1%) (Table 1). The majority (146, 83.0%) identified themselves as Muslim, but very few were diabetes patients themselves (11, 6.3%). In-patient (72, 40.5%) and ambulatory care (103, 57.8%) practice sites were similarly represented.

**Table 1:** Subject demographics (n = 178,%)

	
Gender (n,% female)*	79 (45.4)
Age, years (mean, SD)	35.5 (8.1)
Self-identified as Muslim**	146 (83.0)
Self-identified as having diabetes**	11 (6.3)
Highest pharmacy degree (n,%)	
Baccalaureate	156 (87.6)
Masters	13 (7.3)
Doctorate (PhD or PharmD)	7 (3.9)
Year of highest pharmacy degree (n,%)**	
Less than 2 years ago	14 (8.0)
2–5 years ago	21 (12.1)
6–10 years ago	45 (25.9)
11–15 years ago	53 (30.5)
More than 15 years ago	41 (23.6)
Current pharmacy practice (n,%)**	
Community	65 (36.5)
Clinic (private or public)	38 (21.3)
Hospital (private or public)	72 (40.5)
Other	3 (1.7.)

*n = 175 respondents; **n = 176 respondents.

More pharmacists estimated usual daily interactions occurring with diabetes patients (91, 51.1%) than in specifically the 1–2 months directly in advance of and during Ramadan (51 each, 28.7%). However, estimates of weekly interaction leading up to and during Ramadan increased (46, 25.8% compared to 69, 38,7% and 62, 34.8%). Only very few (4, 2.2%) reported never having any level of interaction with diabetes patients at anytime throughout the year. There was no statistically significant difference in frequency of interactions with diabetes patients during Ramadan between the outpatient and inpatient pharmacists (χ^2^ (1, N = 178) = 2.23, p = 0.158). When interacting with patients of varying risks for complications during Ramadan, very few indicated they would advise low (5%) or moderate (9%) risk diabetes patients against fasting, but 30% and 60% would discourage those diabetes patients with high or very high risk profiles, respectively. However, if appropriate dose adjustments and enhanced self-monitoring were in place for high and very high risk diabetes patients who still insisted on participating in Ramadan, the proportion of pharmacists who still discouraged fasting decreased to 20% and 38%, respectively.

The most popular resources for diabetes management during Ramadan among the respondents were the internet (94, 52.8%); published clinical practice guidelines (80, 44.9%), and personal opinions or experiences (50, 28.1%). Use of materials distributed by local organizations (such as the Qatar Diabetes Association) was low (32, 18%). Twenty percent were aware of and had read the American Diabetes Association (ADA) Consensus document on fasting during Ramadan, but only 8.3% had done so for the decree of the Organization of Islamic conference, a guidance article arising from symposia between the Islamic Organization for Medical Sciences and the International Islamic Fiqh Academy.

One hundred and thirty seven respondents completed all knowledge questions pertaining to diabetes management during Ramadan (Table 2). The mean score was 30 (standard deviation 8) and overall respondent knowledge category was fair (101, 73.7%). Few pharmacist answers reflected a “good” score (10, 7.3%), but more pharmacists than this scored “poor” (26, 19%). Overall, pharmacists performed best when characterizing patient-specific fasting risks (84% of responses were correct) while difficulty was observed for identifying appropriate patient advice and education (40% of responses were correct). Only 43% of correct responses were given for the questions pertaining to drug dosing adjustment during Ramadan fasting (over 90% chose incorrect strategies for altering insulin). There was no statistically significant relationship between practice site and level of knowledge (χ^2^ (1, N = 137) = 0.280 p = 0.644). When logistic regression was performed, no association between knowledge and either frequency of interaction with diabetes patients during Ramadan (p = 0.594) or years of practice experience (0.185) with knowledge was found.

**Table 2:** Select knowledge questions regarding fasting diabetes patient care

**Question**	**Answer**	**Correct response (n = 137,%)**
Fasting diabetes patient risk stratification		
Type 1 diabetes patient	High risk	83 (60.5)
Well-controlled patients using metformin	Low risk	89 (64.9)
Well-controlled patients using sulfonylurea	Moderate risk	44 (32.1)
Patients with history of recurrent hypoglycemia	High risk	107 (78.1)
Pregnant patient	High risk	84 (61.3)
Elderly patients in ill health	High risk	90 (65.7)
Fasting diabetes patient advice		
This patient must be referred to a physician for assessment before embarking on Ramadan fast	All patients	12 (8.7)
This patient can safely fast with appropriate dose adjustment and frequent monitoring	Moderate and low risk patients	86 (62.8)
This patient must be advised not to fast	High risk patient	53 (38.7)
Diabetes patients should break their fast if blood glucose drops to equal or less than 60 mg/dL (3.3 mmol/L)	True	107 (78.1)
Diabetes patients should break their fast if blood glucose reaches 70 mg/dL (4 mmol/L) in the first few hours after the start of the fast	True	75 (54.7)
Fasting diabetes drug dosing adjustment for fasting		
A 55 y.o. with type 2 diabetes takes metformin 500 mg po three times daily	Take 1 g after Iftar meal & take 500 mg after Suhur meal	69 (50.4)
A 35 y.o. with type 2 diabetes takes sitagliptin po 100 mg once daily	Take 100 mg after Iftar meal	82 (59.8)
A 35 y.o. with type 1 diabetes is well-controlled taking 20 units of insulin (Lantus^®^) at 9 a.m. and 15 units of insulin (Actrapid^®^) before each meal	Adjust insulin Lantus to 16 Units with Suhur meal	10 (7.3)

Pharmacists identified several practical barriers to their ability to guide diabetes management during Ramadan, including inadequate privacy (81, 63%); high workload (51, 39%) and patient volume (66, 51%); patient time constraints (59, 44%) or rejection of counseling (28, 21%); and language barrier (41, 31%). They also faced health system obstacles, which include lack of access to patient medical records (60, 50%) or reimbursement for services (51, 39%). While 24 (20%) were concerned about offering advice that may inadvertently contradict the physician, 107 (88%) disagreed with the offered statement that it was not their job as a pharmacist. In fact 117 (92.9%) believed pharmacists were qualified to provide counseling (the same proportion assigned to physicians). This perceived education ability declined when dietitians (88, 51%) and nurses (46, 27%) in turn were considered.

Despite such issues, most respondents were motivated to participate in the management of diabetes patients during Ramadan and to collaborate in determination of patient eligibility for safe fasting (105, 85%); adjusting medication dosage and timings (106, 86%) and developing educational materials (115, 93%) (Table 3). These pharmacists expressed positive attitudes towards such activities and agreed perceptions and visibility of pharmacist would be enhanced (117, 94%), patient service satisfaction improved (118, 94%) and overall role of pharmacist in multidisciplinary care strengthened (114, 91%).

**Table 3:** Pharmacist interest in contributing to specific aspects of fasting diabetes patient care during Ramadan (n = 125,%)

	**Strongly agree or agree**	**Neither agree or disagree**	**Strongly disagree or disagree**
Determining patient risk category for fasting using available expert recommendations	104 (83.2)	3 (2.4)	4 (3.2)
Referring all diabetes patients for medical assessment by their physician 2 months before Ramadan	87 (63.5)	18 (14.4)	18 (14.4)
Recommending dose adjustment for diabetes patients taking insulin	104 (83.2)	20 (16)	19 (15.2)
Advising the adjustment of dose and time of administration of oral diabetes agents	107 (85.2)	7 (5.6)	9 (7.2)
Developing educational material regarding management of hypo- or hyperglycemia during Ramadan	115 (92)	5 (4)	2 (1.6)

## Discussion

While few studies exist assessing health professional attitudes towards Ramadan fasting of diabetes patients , this is the first evaluation of how such patients may be managed by pharmacists within a Middle Eastern context, which is noteworthy given an estimated 20% of the world’s 1.5 billion Muslims live in this region [10,19-21].

The majority of responding pharmacists exhibited fair overall knowledge regarding education and management of fasting diabetes patient. While it would follow that Muslim health professionals or pharmacists with diabetes themselves would be more knowledgeable due to personal acumen, the number of diametric respondents in our sample were too small to make such comparisons. Similarly, we could not identify any association between practice site, experience, or self-reported frequency of diabetes patient interactions on knowledge.

Deficiencies in correctly answering questions pertaining to diabetes drug dosing and administration adjustment by pharmacists is concerning and inadequate medication counseling has also been reported among physicians. A small study of 101 French general practitioners identified few (53%) would adjust insulin dosing for type 1 diabetes patients during Ramadan and most who would admitted not offering specific instructions to the patient. Only 12% of these physicians thought to recommend checking blood glucose more frequently [10]. A Canadian focus group study including 22 health care professionals found these clinicians expressing uncertainty towards counseling Muslim diabetes patients during Ramadan, especially in the presence of divergent patient beliefs [19].

However, a number of published resources exist to guide management of diabetes patients wishing to participate in the Ramadan fast. Only a small proportion of our cohort of pharmacists were familiar with the two pivotal guidelines we chose, but they could have been acquainted with other suitable references. Indeed, the most commonly cited resource for these pharmacists was the internet, but we are unable to elaborate on the nature, appropriateness or quality of information they purported to locate.

Identified gaps in knowledge and inconsistencies in care should be viewed as a pressing opportunity for correction as there is evidence that structured education programs reduce poor outcomes among fasting diabetes patients. Multidisciplinary small group education regarding drug dosing, glucose monitoring, diet and physical activity during Ramadan has been shown to decrease hypoglycemic and hyperglycemic events in participants [22,23]. Specific medication management advice for oral glucose lowering therapies, insulin, and other parenteral agents (e.g. GLP-1 agonists, amylin analogs) is paramount for safe fasting by diabetes patients. If such programs are to be rightly pursued in the Middle Eastern region, our study indicates that continuing education initiatives must be in place for health care providers and especially pharmacists, to facilitate communication of accurate and practical health advice. Unfortunately, patient enrolment capacity and resources available to offer structured multidisciplinary education programs falls well below needs of the diabetes population [24]. Studies of Muslim patients living in North America, Europe, and Australia describe very low rates of information provided to them regarding overall health or specific medication management strategies during the Ramadan fast [10-12,19,20]. Pharmacists are in a unique position to serve as a source of information. Previous North American study estimates diabetes patients see a pharmacist seven times for every one encounter with another health care provider and numerous studies have demonstrated pharmacist interventions translating into improvements in diabetes outcomes [25]. It is not only diabetes patients who would benefit from greater access to fasting advice and care as a number of other patient groups at-risk for complications choose to participate in Ramadan [26,27]. Similarly, fasting among Muslim populations is not restricted to the holy month of Ramadan, as religious and personal situations dictate periods of fasting during other times throughout the year.

Qatar pharmacists responding to this survey expressed positive attitudes and motivation to engage patients in diabetes care during Ramadan. They also demonstrated realistic expectations about these patients’ desire to honour their religious obligations, unlike those of health professionals found in previous studies. Over half (55%) of a small group of Muslim Turkish patients (n = 36) residing in Belgium were advised against fasting and over half (54%) of French general practitioners reported they discourage fasting among their diabetes patients [10,12]. Muslim diabetes patients in Australia described experiences whereby physicians failed to acknowledge the importance of participating in the Ramadan fast and therefore abandoned efforts to consult them regarding this specific care [20]. Our findings may be attributed to this being the first study in the Middle East among mostly Muslim health care providers, as well as our stratifying diabetes patients according to defined risk categories when exploring pharmacists recommendations to such patients for participating in the Ramadan fast.

Practical barriers to Qatar pharmacist provision of diabetes care may reflect those of other pharmacists elsewhere in the region that are slowly being addressed. Government-initiated consultation and assessment has identified that the existing primary care system in Qatar does not play a sufficiently strong role in preventing, monitoring, and treating diseases. In response, key initiatives exist within the country’s recently launched National Health Strategy to overhaul the health care system with a six-year plan to address several deficit areas, which notably includes medication and patient safety [16]. In addition, health care policy leaders formally identify the ideal model of health care delivery must be integrated, with different health professional providers working cohesively and a complementary mandate issued to encourage greater roles for pharmacists. This multidisciplinary approach is consistent with a standard of care endorsed by the International Diabetes Federation (IDF). Similarly, a number of studies have demonstrated pharmacist interventions translates into improvements in diabetes outcomes [28,29].

Several important limitations to this study merit consideration. The absence of a formal national pharmacist registry means our study sample is incomplete. Non-response error (occurring with survey response rates <60%) compromises accuracy of our conclusions. Although this may further contribute to selection bias and restrict the generalizability of our study findings, this response rate is consistent with other pharmacist surveys conducted in Qatar. Higher response rates have been reported in similar surveys in instances where investigators visited practice sites (especially community pharmacies) for face-to-face interviews with the pharmacists, but this was not feasible given our study’s time frame and available resources. Generally speaking, those pharmacists participating in the survey demonstrated an inherent bias towards sharing opinions on diabetes patient care during Ramadan and so important views of pharmacists who were not inclined to answer the questionnaire have been overlooked by this specific research methodology; therefore, our findings regarding knowledge and attitude may be overestimations and the barriers to reporting underestimations. Another limitation concerns the survey tool itself. The questionnaire was not previously validated, but this is not unusual for the preliminary and exploratory nature of our study [30]. However, it was developed considering related published qualitative data and we conducted a pilot study to adapt it to pharmacists’ comprehension. As for any self-administered questionnaire, accurate responses may have been impaired by the inability to clarify question intent with a web-based survey and because completion was unsupervised and at the respondent’s leisure, pharmacists could have suspended completing the questionnaire in order to look up certain answers.

## Conclusions

There is a dearth of published international experience related to multidisciplinary care of diabetes patients during Ramadan. This study is the first to describe aspects of fasting diabetes patient care during Ramadan by pharmacists in Qatar. Pharmacists can play a pivotal role in ensuring patients seek medical assessment in advance of Ramadan to plan their fast and also to advise on dosing adjustment and glucose management. Educational programs are necessary to improve pharmacist knowledge in the provision of accurate advice for fasting diabetes patients.

## Competing interests

The authors declare they have no competing interests.

## Authors’ contributions

KW conceived the project, organized, analyzed and interpreted the data and wrote the manuscript. KA and ER collected and interpreted data and reviewed the manuscript. All authors read and approved the final manuscript.

## Pre-publication history

The pre-publication history for this paper can be accessed here:

http://www.biomedcentral.com/1472-6963/14/117/prepub
